# Heterologous expression of *Plasmodium vivax* apical membrane antigen 1 (PvAMA1) for binding peptide selection

**DOI:** 10.7717/peerj.3794

**Published:** 2017-09-13

**Authors:** Ching Hoong Chew, Yvonne Ai Lian Lim, Kek Heng Chua

**Affiliations:** 1 School of Biomedicine, Faculty of Health Sciences, Universiti Sultan Zainal Abidin, Kuala Nerus, Terengganu, Malaysia; 2 Department of Parasitology, Faculty of Medicine, University of Malaya, Kuala Lumpur, Malaysia; 3 Department of Biomedical Science, Faculty of Medicine, University of Malaya, Kuala Lumpur, Malaysia

**Keywords:** *Plasmodium vivax*, Recombinant protein expression, Apical membrane antigen 1 (AMA1), Binding peptide, Phage display, *In silico* peptide docking

## Abstract

**Background:**

*Plasmodium* is an obligate intracellular parasite. Apical membrane antigen 1 (AMA1) is the most prominent and well characterized malarial surface antigen that is essential for parasite-host cell invasion, i.e., for sporozoite to invade and replicate within hepatocytes in the liver stage and merozoite to penetrate and replicate within erythrocytes in the blood stage. AMA1 has long served as a potent antimalarial drug target and is a pivotal vaccine candidate. A good understanding of the structure and molecular function of this *Plasmodium* protein, particularly its involvement in host-cell adhesion and invasion, is of great interest and hence it offers an attractive target for the development of novel therapeutics. The present study aims to heterologous express recombinant* Plasmodium* AMA1 ectodomain of *P. vivax* (rPvAMA1) for the selection of binding peptides.

**Methods:**

The rPvAMA1 protein was heterologous expressed using a tag-free Profinity eXact^TM^ system and codon optimized BL21-Codon Plus (DE3)-RIL *Escherichia coli* strain and further refolded by dialysis for renaturation. Binding peptides toward refolded rPvAMA1 were panned using a Ph.D.-12 random phage display library.

**Results:**

The rPvAMA1 was successfully expressed and refolded with three phage-displayed dodecapeptides designated as PdV1 (DLTFTVNPLSKA), PdV2 (WHWSWWNPNQLT), and PdV3 (TSVSYINNRHNL) with affinity towards rPvAMA1 identified. All of them exhibited positive binding signal to rPvAMA1 in both direct phage assays, i.e., phage ELISA binding assay and Western blot binding assay.

**Discussion:**

Phage display technology enables the mapping of protein-protein interactions based on a simple principle that a library of phage particles displaying peptides is used and the phage clones that bind to the target protein are selected and identified. The binding sites of each selected peptides toward PvAMA1 (Protein Data Bank, PDB ID: 1W8K) were *in silico* predicted using CABS-dock web server. In this case, the binding peptides provide a valuable starting point for the development of peptidomimetic as antimalarial antagonists directed at PvAMA1.

## Introduction

Human malaria is a life-threatening, highly infectious parasitic disease caused by the intracellular, protozoan parasites, *Plasmodium* species, including *P. vivax*, *P. falciparum*, *P. ovale*, *P. malariae* and, *P. knowlesi*. Amongst the five human malaria parasites, *P. vivax* is the most prevalent and geographically widespread species, with approximately 35% of the world’s population at risk (Gething et al., 2012). In 2015, *P. vivax* morbidity accounted for approximately 8.5 million global malaria cases (212 million), mainly concentrated outside the African continent. Most *P. vivax* malaria cases occur in the WHO South-East Asia Region (58%), followed by the Eastern Mediterranean Region (16%) and the African Region (12%). Four countries (Ethiopia, India, Indonesia, and Pakistan) accounted for 78% of *P. vivax* cases and 81% of estimated deaths due to *P. vivax* malaria (WHO, 2016). The ability to persist in dormant hypnozoites form during liver stage is a specific characteristic of *P. vivax*, which has the tendency to cause multiple clinical relapses over months to years following a primary infection (White, 2011). Although in many cases, *P. vivax* infection has been neglected as a benign infection, *P. vivax* infection does cause serious clinical manifestations in some circumstances, including severe anaemia and malnutrition, multi-organ involvement such as acute lung and/or kidney injuries, respiratory distress, coma, and even death, especially for drug resistance strains (Anstey et al., 2012; Baird, 2013). Generally, global malaria control and elimination strategies are mainly focused on the more pathogenic and deadly falciparum malaria, in which early diagnosis, prompt and effective treatment is a priority. However, these strategies are not applicable to *P. vivax* cases because this species tolerates a wider range of environmental conditions. Besides that, early appearance of gametocytes in infected human before clinical symptoms are apparent and a shorter development cycle in the *Anopheles* vector have complicated the elimination process of *P. vivax*. For these reasons, availability of an effective vaccine that provides protection and prevents transmission would be a valuable tool in the efforts to eliminate *P. vivax* (Mueller, Shakri & Chitnis, 2015; WHO, 2015).

*Plasmodium* is a member of the *Apicomplexa* phylum which has a defining characteristic of possessing a set of organelles collectively known as apical organelles localized at the apical end of the parasite. The apical complex which includes secretory organelles, i.e., micronemes and rhoptries lie within the polar ring and these organelles are highly regulated and expressed in some vital stages of the parasite’s life cycle. Nowadays, a number of apical proteins have been implicated in the invasion process and amongst these malarial surface proteins, apical membrane antigen 1 (AMA1) is one the most well characterized malaria surface antigen that is crucial for host cell invasion. Generally, AMA1 is a micronemal protein expressed abundantly in sporozoites responsible for hepatocytes invasion as well as merozoites at the end of the tissue schizogony (pre-erythrocytic stage) and erythrocytic schizogony (erythrocytic stage) responsible for erythrocytes invasion, therefore it offers the potential for the development of therapeutics or vaccines acting against these two critical stages (Healer et al., 2002; Silvie et al., 2004). AMA1 is a type I integral membrane protein, build-up of a prosequence domain, an ectodomain (ectoplasmic region), a single transmembrane domain, and a small C-terminal cytoplasmic domain. The ectoplasmic region of AMA1 comprises of 16 invariant cysteine residues that are cross linked and folded into eight pairs of conserved disulfide bonds, which are dispersed and they define the ectodomain into three distinct subdomains, i.e., domain I (DI), domain II (DII), and domain III (DIII). The eight disulfide bonds are essential for structure stability and functionality of the AMA1 protein (Hodder et al., 1996). Despite AMA1 being a low abundance malaria surface antigen, it represents the most prominent immunogen that is able to stimulate strong immune response in both human and animal models, therefore widely regarded as a potent target of antimalarial drugs and pivotal malaria vaccine candidate (Remarque et al., 2008; MacRaild et al., 2011). Numerous studies have confirmed that monoclonal and polyclonal antibodies and peptides derived from or with affinity to AMA1 of *Plasmodium* block the entry of parasite into host cell *in vitro* or protect against blood stage growth *in vivo* (Keizer et al., 2003; Wang et al., 2014; Vulliez-Le Normand et al., 2015). In general, formation of moving junction between merozoite and erythrocyte is the key role for successful parasite host cell invasion and the mechanism involve the interaction between the conserved hydrophobic groove in DII loop of AMA1 and the conserved rhoptry neck protein 2 (RON2) loop (Bargieri et al., 2013). Prior to invasion, AMA1 protein that is initially stored in micronemes is translocated to the surface of merozoite, while RON2 is secreted from the rhoptries and transferred to the erythrocyte surface (Vulliez-Le Normand et al., 2012). Even though the exact molecular function of AMA1-RON2 complex remains a matter of debate, it is clear that inhibition of the AMA1-RON2 interaction by various agents effectively disrupts invasion and thus validates that AMA1 is a viable therapeutic target (MacRaild et al., 2011; Bargieri et al., 2014; Wang et al., 2014).

Phage display technique is a powerful tool that allows large scale screening and identifying protein-protein, protein-peptide, and protein-DNA interactions (Mullen et al., 2006). Phage display technology is comparably faster and a relatively inexpensive interaction assay compared with three dimensional (3D) structuring technologies. In an attempt to provide insight into the biological function and immunological properties, which lies behind the targeted *Plasmodium* proteins, many studies have been done using phage display libraries and have been reviewed and summarized by Lanzillotti and Coetzer (Lanzillotti & Coetzer, 2008). In the present study, we therefore aimed to investigate and identify the possible binding peptides, which are highly bond to recombinant AMA1 ectodomain protein of *P. vivax* (rPvAMA1) using a phage display random dodecapeptide library. The study was initiated with heterologous recombinant protein expression using a tag-free Profinity eXact™ system in codon optimized host, BL21-Codon Plus (DE3)-RIL *Escherichia coli* strain to produce sufficient starting material for the use in the biopanning process against refolded rPvAMA1.

## Materials and Methods

### Template DNA of *Plasmodium vivax*

DNA from a clinical specimen with PCR-confirmed *P. vivax* infection was used as the template for recombinant protein expression study (Chew et al., 2012). Sequencing of the 18S ssurRNA clone confirmed that this isolate belongs to the *P. vivax* strain Sal-1 (GenBank accession number: U03079).

### Construction of pPAL7-PvAMA1 plasmid

The tag-free Profinity eXact™ system (Bio-Rad, Hecules, CA, USA) was used to express the recombinant PvAMA1. PvAMA1-forward (ATATATACTAGTGGGCCTACCGTTGAGAGRAGC)  and PvAMA1-reverse (AGACAAGGATCCTTATAGTAGCATCYGCTTGTTCGA) primers were designed with *Spe*I and *Bam*HI restriction enzymes recognition sites (underline) to adapt the PvAMA1 ectodomain gene for directional insertion into pPAL7 expression vector (Bio-Rad). DNA fragment was amplified using TaKaRa PCR Thermal Cycler Dice™ (Takara Bio, Kusatsu, Shiga Prefecture, Japan) and KOD Hot Start DNA polymerase (Novagen®, Merck KGaA, Darmstadt, Hesse, Germany). Both PvAMA1 fragment and supercoiled pPAL7 plasmid (Bio-Rad, Hercules, CA, USA) were double digested with *Spe*I (5′-A/CTAGT-3′) and *Bam*HI (5′-G/GATCC-3′) according to the manufacturer’s instructions. The gel purified restriction enzymes digested-PCR product and pPAL7 vector were ligated using Ready-To-GO™ T4 DNA Ligase (Amersham Biosciences, Little Chalfont, Buckinghamshire, UK) and then transformed into C-Max5α *E. coli* competent cell. The C-Max5α *E. coli* that carried the desired plasmid was confirmed by colony PCR and sequenced using T7 promoter (F) and T7 terminator (R) primers were re-transformed into codon-optimized expression host, i.e., BL21-Codon Plus (DE3)-RIL *E. coli* (Stratagene) as per manufacturer’s instructions. The successful clones were selected onto LB agar plates supplemented with 100 µg/ml of ampicillin and 34 µg/ml of chloramphenicol to select pPAL7 plasmid and pACYC-based plasmid that confers chloramphenicol resistance, respectively. Successful colonies were further confirmed by colony PCR and sequencing. The expression *E. coli* clone was stocked in LB broth containing 100 µg/ml ampicillin, 34 µg/ml chloramphenicol and 15% (v/v) glycerol and stored at −80 °C until further application.

### Expression of rPvAMA1 protein

Ten ml of LB agar supplemented with 100 µg/ml ampicillin and 34 µg/ml chloramphenicol was inoculated with 5 µl of frozen stock culture and grown overnight as seed culture. One ml of overnight seed culture was added to 100 ml of 2× YT medium containing 100 µg/ml ampicillin and 34 µg/ml chloramphenicol and incubated at 37 °C with 225 rpm shaking for ∼3 hrs (until turbidity reached OD_600_∼0.6). Protein expression was induced with 0.5 mM IPTG and further incubation for another 3 hrs at 25 °C with vigorous agitation at 250 rpm. The bacteria cells were harvested by centrifugation at 4,000 × g for 20 min at 4 °C. The cell pellet was kept at −80 °C for further recombinant protein isolation.

### Isolation of Profinity eXact-tagged PvAMA1

The cell pellet was resuspended in 5 ml of B-PER® in phosphate buffer (Pierce, Rockfield, IL, USA) in the presence of 1× Calbiochem® Protease Inhibitor Cocktail Set I (Novagen), 200 µg/ml of lysozyme (Novagen), and 10 U/ml of Benzonase (Novagen). Lysate was disrupted using 25 Gauge syringe needle followed by vortexed for a minute, and incubation at room temperature for 20 min with shaking at 40 rpm on an orbital rotary (Major Sciences, Saratoga, CA, USA). The protein fractions obtained along cells lysis process were analyzed with reduced 12% SDS-PAGE to determine the solubility fraction of the recombinant Profinity eXact-tagged PvAMA1 (∼60 kDa). The solubility assay and Western blot assay indicated that the target protein was expressed in inclusion body (IB) and therefore the IB portion was collected and stored at −80 °C until further purification steps.

### SDS-PAGE and Western blot analyses

The solubility, expression level, and purity of fusion protein, i.e., rPvAMA1 were analyzed using Coomassie-stained 12% SDS-PAGE under reduced conditions and colorimetric Western blotting assay using the anti-eXact monoclonal antibody (Bio-Rad) and visualization with horseradish peroxidase (HRP) using Opti-4CN substrate kit (Bio-Rad). The Western blot results were documented with ImageScanner III (GE Healthcare).

### Purification of inclusion body

Inclusion body (IB) was resuspended in 5 ml of wash buffer 1 (B-PER buffer, 200 µg/ml lysozyme, 1% (v/v) Triton X-100, 5 mM EDTA, 5 mM DTT, 1 M urea) using 25 Gauge syringe needle followed by incubation at room temperature for another 20 min with shaking at 40 rpm on an orbital rotary (Major Sciences). A total of 15 ml pre-chilled IB wash buffer 2 (1:10 diluted B-PER, 1% (v/v) Triton X-100, 5 mM EDTA, 5 mM DTT, 1 M urea) was added to the suspension and mixed by vortexing for 1 min followed by centrifugation at 16,000 × g for 15 min at 4 °C. The pelleted IB was then resuspended and washed with 15 ml of IB wash buffer 3 (1:10 diluted B-PER) followed by centrifugation at 16,000 × g for 15 min at 4 °C to remove excess Triton X-100 from the pellet. The washed IB pellet becomes whiter as the purity increased.

### On-column isolation and purification of tag-free rPvAMA1

Purified IB was resuspended in IB solubilization buffer (100 mM sodium phosphate, pH 7.2, 4 M urea, 5 mM DTT, 1 mM EDTA) and incubated overnight at 4 °C. Suspension of protein was then centrifuged at 15,000 × g for 30 min at 4 °C. Supernatant containing denatured tagged rPvAMA1 was collected and filtered through 0.45 µm pore size cellulose acetate membrane filter (Sartorius Stedim Biotech, Aubagne, Marseille, France) prior to recombinant protein purification to prevent clotting of the Profinity eXact™ purification cartridge (Bio-Rad). Generally, the denatured purification protocol was adapted from manufacturer’s instructions that based on soluble protein purification, with inclusion of 4 M urea (denaturant) in both bind/wash and elution buffers along purification procedures. The purity of the tag-free rPvAMA1 was determined using reduced 12% SDS-PAGE and then stored at −80 °C until further manipulation.

### Renaturation of purified rPvAMA1

The purified tag-free rPvAMA1 in elution buffer (4 M urea, 1× of Calbiochem® Protease Inhibitor Cocktail Set I, 100 mM sodium phosphate, 100 mM sodium fluoride, pH 7.2) was concentrated to ∼2 mg/ml using Vivaspin 20 concentrator fitted with 5 kDa MWCO PES membrane (Sartorius Stedim Biotech) as per manufacturer’s instructions. The concentrated protein was treated with 5 mM DTT and 1 mM EDTA then incubated at room temperature for one hour. Prior to refolding, the reduced protein was buffer exchanged to refolding buffer (1 mM reduced glutathione (GSH), 0.25 mM oxidized gluthathione (GSSG), 4 M urea, 50 mM Tris phosphate, pH 8) using 10 kDa MWCO Zeba Desalting Column (Pierces Thermo Scientific).

Protein refolding was performed by dialysis method using 3 ml Slide-A-Lyzer Dialysis Cassettes with 10 kDa MWCO (Pierces Thermo Scientific, Waltham, MA, USA). Protein sample in the cassette was dialyzed against two changes of 400 ml of dialysis buffer (50 mM Tris phosphate, 0.4 M L-arginine, 1 mM EDTA, pH 8) with stepwise decrease in urea concentration, i.e., 2 M and 1 M at 4 °C with stirring for four hours in each change, which allows the protein to refold optimally. After the last change, the protein solution was further dialyzed overnight at 4 °C against sample buffer without urea (25 mM Tris phosphate, 50 mM NaCl, pH 8), which is compatible to downstream application, i.e., biopanning. The efficiency of refolding condition was monitored by measuring the protein aggregation by simple visual inspection and turbidity analysis and reduced/non-reduced SDS-PAGE (Burgess, 2009). In general, turbidity of the refolded rPvAMA1 protein was assessed by measuring the OD at 390 nm using NanoPhotometer™ (IMPLEN, Munich, Bavaria, Germany) for refolding screening. The turbidity was determined by subtracting the apparent OD reading of the sample solution to the blank sample buffer (25 mM Tris phosphate, 50 mM NaCl, pH 8) and the rPvAMA1 was taken to be soluble at OD < 0.05. Evidence of successful protein refolding was further analyzed through SDS-PAGE under reducing and non-reducing conditions in the presence or absence of β-mercaptoethanol, respectively in the protein loading buffer.

### Protein identification by LC-MS/MS

Recombinant PvAMA1 ectodomain was electrophoresed using 12% SDS-PAGE under non-reduced conditions followed by staining with Coomassie Blue. The band of interest was excited from the gel and reduced (20 mM Tributylphosphine), alkylated (40 mM Iodoacetamine), and in-gel trypsin digestion, and then sent for liquid chromatography tandem mass spectrometry (LC-MS/MS) analysis (Monash University Malaysia) for protein identification. The LC-MS/MS was performed using Agilent 6520 Accurate-Mass Q-TOF LC/MS system (Agilent Technologies, Santa Clara, CA, USA). Peptide ions were analyzed with GPS Explorer software (Applied Biosystems, Foster City, CA, USA) using MASCOT™ Database.

### Bacteria stain maintenance and phage amplification

The Ph.D.-12 random phage display library was purchased from New England Biolabs and biopanning assay was performed accordingly to manufacture’s recommendations. The *E. coli* ER2738 was cultured on LB plate containing 20 µg/ml of tetracycline and incubated overnight at 37 °C. The plate was then wrapped with parafilm and kept at 4 °C in the dark until needed. The *E. coli* culture for phage infection and propagation was prepared freshly by inoculating a single colony into LB broth containing 20 µg/ml of tetracycline and incubated at 37 °C with a vigorous shaking at 250 rpm until mid-log-phase, OD_600_ ∼ 0.6. Phages were amplified by infecting a mid-log-phase *E. coli* culture and shaking overnight at 37 °C in LB medium containing 25 μg/ml tetracycline. The supernatant was twice clarified by pelleting the cells for 20 min at 4,000 × g at 4 °C and 20% (w/v) of PEG/2.5 M NaCl was added to precipitate the phage. The amplified phages were allowed to precipitate at 4 °C for 2 hrs before being centrifuged at 12,000 × g for 15 min at 4 °C. Phage pallet was resuspended in 200 µl of TBS and stored at −20 °C under equal volume of glycerol and 0.02% NaN_3_.

### Biopanning of the phage display library

Three rounds of panning were performed on refolded rPvAMA1. Biopanning was carried out using Costar high binding, flat bottom 96 well microtiter plate (Corning Incorporated, New York, NY, USA) according to the direct target coating method as described in instruction manual. In general, well was coated with rPvAMA1 (50 µg) in coating buffer (0.1 M NaHCO_3_, pH 8.6), sealed, and incubated overnight at 4 °C. The wells were blocked with 200 µl of blocking buffer (0.5 mg/ml of BSA, 0.1 M NaHCO_3_, pH 8.6, 0.02% (w/v) NaN_3_) and incubated at 4 °C for 1 hr. Following blocking, the wells were washed six times with 300 µl of TBST (TBS plus 0.1% (v/v) Tween 20 for the first round of panning and TBS plus 0.5% (v/v) Tween 20 for the subsequence rounds of panning experiment). Phage library (∼10^11^ particles) was diluted with 100 µl of TBST (TBS plus 0.1% (v/v) Tween 20) and dispensed into coated well and rocked gently at 15 rpm for 1 hr at room temperature. The well was washed thoroughly with TBST for 10 times to remove non-binding phage. Bound phages were eluted using 0.2 M glycine-HCl (pH 2.2) supplemented with 1 mg/ml BSA and neutralized with 1 M Tris–HCl (pH 9.1). One µl of the eluate was titered as described in general M13 phage titering protocol. The rest of the eluate was then amplified and precipitated. Basically, the amplified eluates (10^9^–10^11^ particles) from previous round of biopanning were used for subsequent round of selection.

### Phage titering and plaques forming unit (pfu/ml) determinations

Phage titering was performed to calculate the input and output of phage particles (Chua, Chai & Puthucheary, 2008). One µl of phage was serially diluted to appropriate dilutions in LB broth, ranging from 10 to 10^4^-fold for unamplified phages and 10^8^ to 10^11^ for amplified phages.

### Individual phage amplification for sequencing and peptide binding assays

After the third round of biopanning, 20 phage clones were randomly selected and identified by DNA sequencing. Generally, well separated form of blue colonies were randomly picked from a 1–3 day old titering plate using sterile pipette tip and then amplified in 1 ml log-phase *E. coli* ER2738 culture (one phage per tube). The individual phage clone was amplified and precipitated as routine procedures and the amplified phage stocks were stored at 4 °C until further single stranded DNA isolation for sequencing or peptide binding assays.

### Phagemid single-stranded DNA (ssDNA) extraction and sequencing

Single stranded bacteriophage M13 phagemid DNA was isolated from 500 µl of amplified phage stock as manufacturer’s instructions. The purity of phage ssDNA was measured using NanoPhotometer™ (IMPLEN, Germany) and the concentration was estimated using 0.5 µg of single-stranded M13mp18 DNA ladder (NEB) visualized on 1% (w/v) agarose gel. Ten µl of ssDNA of each phage was sent to 1st Base Malaysia for sequencing service using -96gIII sequencing primer (5′-CCCTCATAGTTAGCGTAACG-3′). The nucleotide sequence of each individual phage variants were then aligned with BioEdit version 7.0.9 Software (Hall, 1999) and a consensus sequences were compared to the sequence of original construct to obtain the translation polypeptide sequence expressed by each of the individual phage.

### Peptides binding assays

The binding affinity of each selected peptide clone to rPvAMA1 was validated using phage ELISA and Western blot binding assays. In general, phage ELISA assay was performed as per manufacturer’s recommendation. Briefly, one row of microwells were coated with rPvAMA1 (100 µg/ml in 0.1 M NaHCO_3_, pH 8.6) and blocked (0.1 M NaHCO_3_, 0.5 mg/ml BSA, 0.02% (w/v) NaN_3_) as biopanning step. Another row of uncoated wells per phage clone to be assayed were also blocked to test for binding of each selected sequence to BSA-coated plastic (background binding). One hundred µl of each of the phage (∼10^9^ in TBST) was filled respectively into one target-coated and one uncoated (blocked) wells and incubated at room temperature for two hours with agitation and washing similar to biopanning steps. The bound phages were detected with HRP-conjugated anti-M13 monoclonal antibody (GE Healthcare) in 1:5,000 dilution in blocking buffer using ABTS™ Chromophore substrate solution (Calbiochem). The reaction was stopped by adding 100 µl of 0.5 M H_2_SO_4_ and the absorbance was read at 405 nm using ELISA iMark™ microplate reader (Bio-Rad). For each phage colony, the signals obtained with and without target protein were compared and the folds of binding were calculated by dividing the absorbance obtained from target protein to the absorbance obtained from BSA.

The binding characteristic of each of the phage peptide was further examined by Western blot binding assay. The rPvAMA1 protein (10 µg) was electrophoresed on 12% SDS-PAGE under non-reducing condition and then blotted onto Whatman Protran nitrocellulose transfer membrane (0.45 µm; Millipore, Billerica, MA, USA). The location of AMA1 protein was confirmed using NOVEX® Reversible membrane protein stain (Invitrogen). The blot was then cut into individual strips. The location of target protein was marked and then destained properly prior to Western blot binding assay. The binding affinity of the individual phage clone (∼10^9^ phage particles) to rPvAMA1 was determined using 1:2,500 HRP-conjugated anti-M13 monoclonal antibody (GE Healthcare) and visualized using Opti-4CN (Bio-Rad) substrate. The results were documented with ImageScanner III (GE Healthcare).

### *In silico* peptide docking

The binding sites of the three peptides, i.e., PdV1, PdV2, and PdV3 to the PvAMA1 were *in silico* predicted using CABS-dock web server (http://biocomp.chem.uw.edu.pl/CABSdock) (Kurcinski et al., 2015; Blaszczyk et al., 2016). The protein PDB code of the PvAMA1, i.e., 1W8K (Pizarro et al., 2005) and each of the peptide sequences were deposited in the web server under three independent peptide docking protocols. At the end of each protocol, the 10 most ranked models (representatives of 10 structural clusters found in simulation) and the quality of each docking models was provided to the user. The quality of the docking models was assessed using ligand RMSD (root-mean-square deviation) as following: RMSD < 3 Å indicates of high-quality prediction; 3 Å  ≤ RMSD ≤ 5.5 Å as medium-quality prediction; RMSD > 5.5 Å as low-quality prediction. In the present study, only one of the top ranked models was analyzed and reported for each of the binding peptide docking protocol. The pairs of peptide/receptor residues with 3.5 Å contact cutoff were then mapped to the native amino acid residues of PvAMA1 (1W8K) and rPvAMA1.

## Results

### Expression of recombinant clones and bioinformatics analysis

The sequencing results indicated that pPAL7-PvAMA1 recombinant clones carried an inserted DNA of 1,338 bp length. The clones were 99% concordance with the published mRNA of the AMA1 ectodomain gene of *P. vivax* (GenBank ref. no.: FJ785007). The ExPASy Translate tool (http://web.expasy.org/translate/) indicated that the inserted coding sequence was in-frame with 446 amino acid residues of AMA1 ectodomain, encompassing the entire region of domain I-II-III. The rPvAMA1 was identical with *P. vivax* AMA1 protein with NCBI accession number ACY68841 (corresponding to amino acids 42–487). The theoretical isoelectric point (*p*I) and molecular weight (MW) of the rPvAMA1 were estimated using ExPASy interface (Compute pI/MW tool; http://web.expasy.org/compute_pi/). The theoretical *p*I/MW of rPvAMA1 was 6.28/51275.85. The Recombinant Protein Solubility Prediction tool (http://biotech.ou.edu/) estimated that 72.5% of rPvAMA1 were probably overexpressed in *E. coli* system in an insoluble form that mainly localized in an IB.

### Recombinant protein expression of Profinity eXact-tag PvAMA1

**Figure 1:**
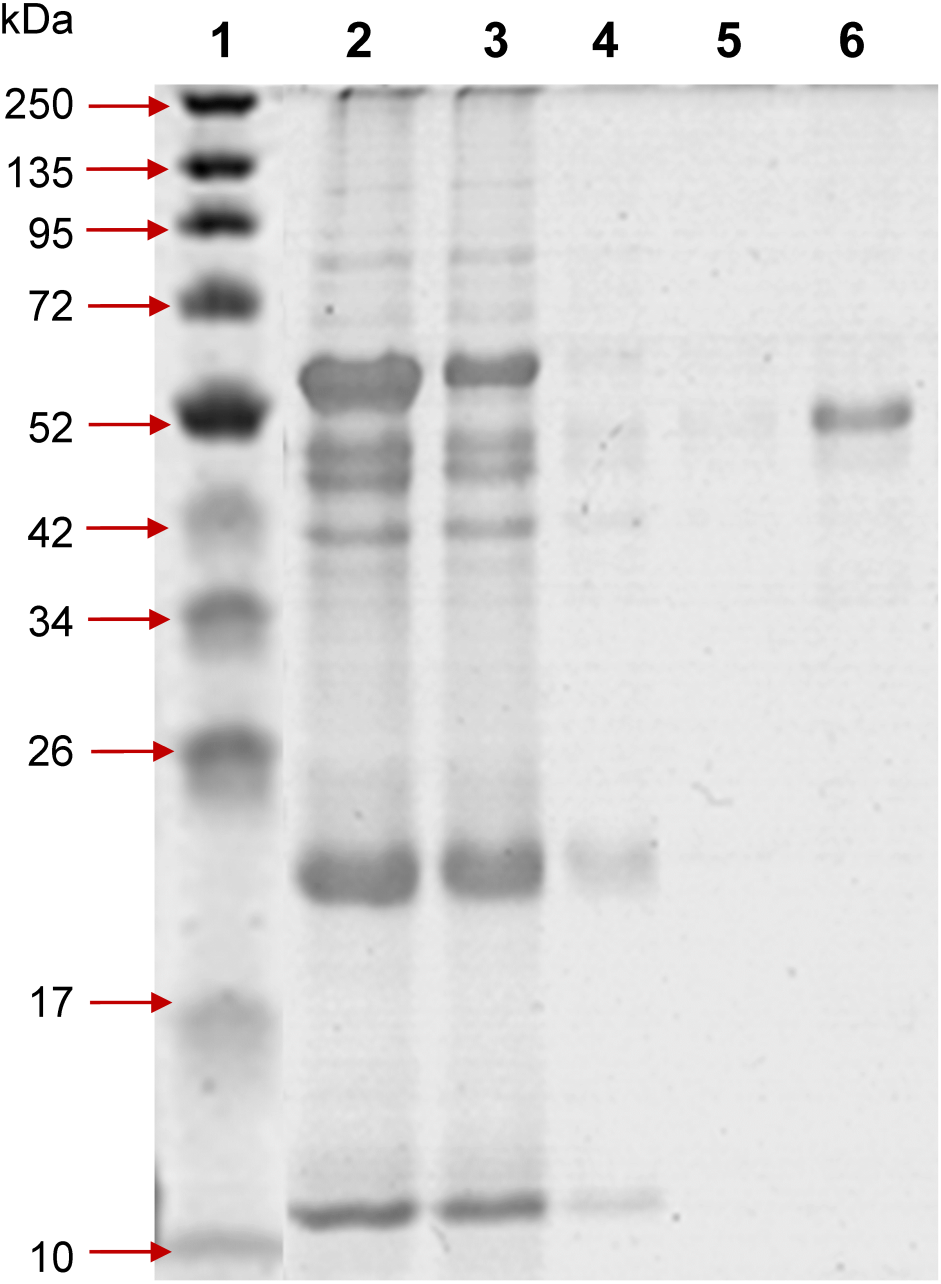
12% SDS-PAGE of profinity eXact tag-free rPvAMA1 purified and eluted using a 5 ml bio-scale Mini™ profinity eXact cartridge under denatured and reduced conditions. Lane 1, protein marker; Lane 2, solubilized IB crude protein; Lane 3, flow-through fraction (excessive target protein and host protein contaminants); Lanes 4 and 5, host protein contaminants from column wash and stringency wash; Lane 6, eluted tag-free rPvAMA1 (∼51 kDa).

Both the SDS-PAGE and Western blot results indicated that the fusion protein, i.e., Profinity eXact-tag PvAMA1was expressed only in IB fraction with the size of an approximately 60 kDa, in concordance with the predicted size. The tag-free rPvAMA1 (∼51 kDa) was successfully on-column isolated and purified from IB fraction under denatured conditions (Fig. 1).

**Figure 2:**
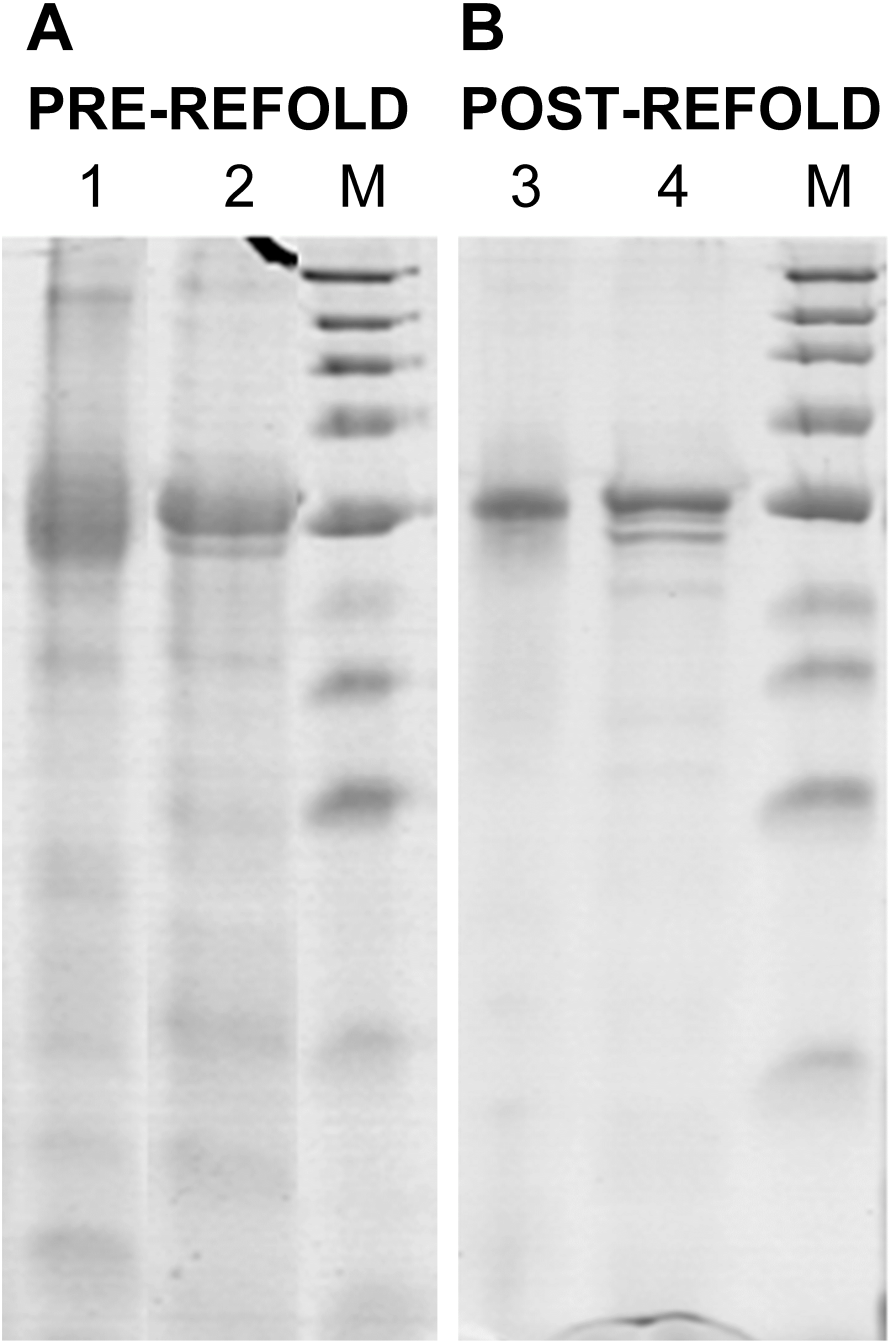
Renaturation of rPvAMA1. 12% non-reduced (Lanes 1 and 3) and reduced (Lanes 2 and 4) SDS-PAGE analysis of pre-refold (A) and post-refold (B) rPvAMA1. Post-refold rPvAMA1 protein is close to apparent homogeneity in ∼51 kDa with disappearance of most disulfide-bonded aggregates. The refolded and non-reduced band (Lane 3) shown to be not diffuse and virtually identical to the refolded and reduced band (Lane 4) indicating most material was refolded successfully. Refolded and non-reduced protein (Lane 3) exhibits a faster mobility than refolded reduced protein (Lane 4) shows the refolding evidence of the rPvAMA1. Shift in mobility upon reduction showed that *β*-mercaptoethanol treatment reduced the expected intramolecular disulfide bonds in the refolded protein and thus suggested that rPvAMA1 produced in this study also contained reduction-sensitive, disulfide-bonded, tertiary structures.

### Renaturation of rPvAMA1

The refolded rPvAMA1 ectodomain protein was taken to be soluble with the apparent OD_390_ reading for sample solution subtracted to blank sample buffer being less than 0.05. After refolding, rPvAMA1 was close to apparent homogeneity in a molecular mass of ∼51 kDa (Fig. 2). Furthermore, there were disappearance of most disulfide-bonded aggregates in post-refold samples and none of dimmers, trimmers, and multimers was observed in post-refolded rPvAMA1 (Lanes 3 and 4). Besides that, the resulting refolded and non-reduced band (Lane 3) was not diffuse and virtually identical to the reduced band (Lane 4) indicating most material was refolded successfully. Figure 2 also shows the refolding evidence of the rPvAMA1, where the refolded and non-reduced protein (Lane 3) exhibits a faster mobility than refolded and β-mercaptoethanol-treated protein (Lane 4). Shift in mobility upon reduction showed that β-mercaptoethanol treatment reduced the expected intramolecular disulfide bonds in the refolded protein and thus suggested that rPvAMA1 produced in this study also contained reduction-sensitive, disulfide-bonded, tertiary structures.

### Biopanning toward refolded rPvAMA1

**Figure 3:**
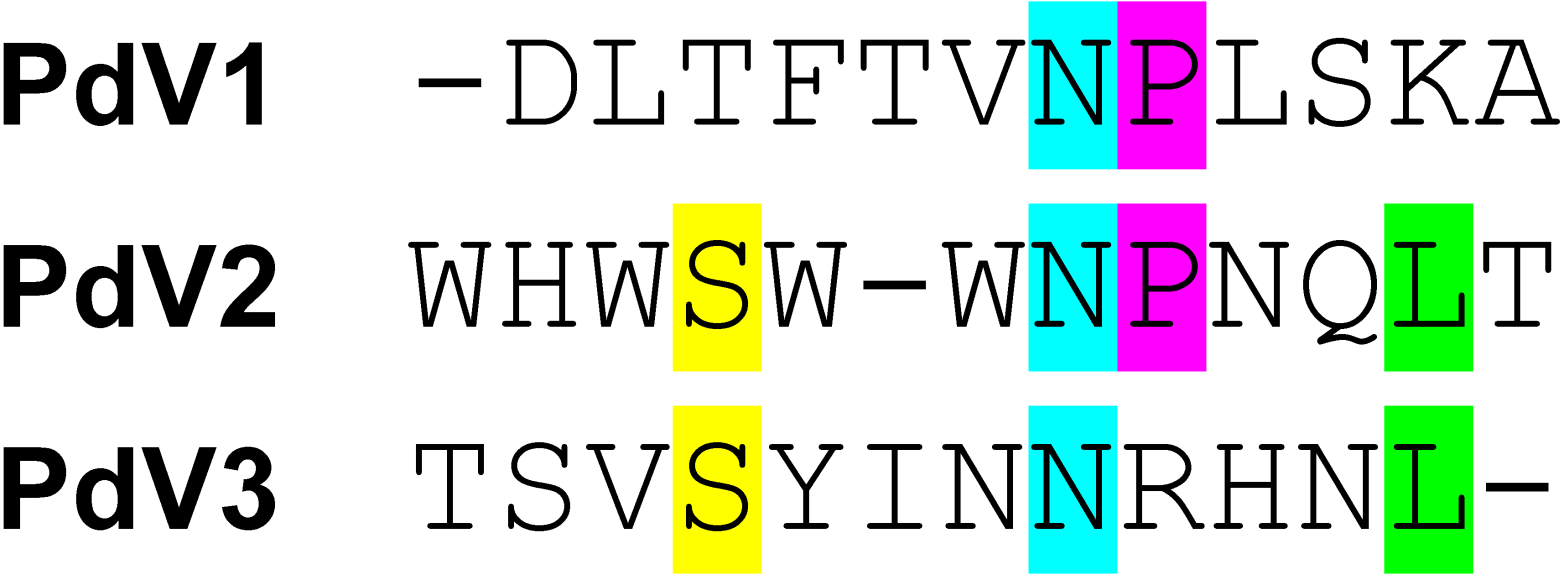
Consensus sequences alignment of selected dodecapeptides obtained after three rounds of biopanning against rPvAMA1. The color bar represented the consensus amino acids located at the same position of consensus motif. Overall, there were a lack of consensus sequence motif found among binding peptides selected from biopanning against rPvAMA1 (PdV1, PdV2, and PdV3). Single Asn (N) residue occurs among three positive binding peptides. One additional consensus sequence of Pro (P) followed after Asn (N) amino acid was found between PdV1 and PdV2. Two additional consensus amino acids, i.e., Ser (S) and Leu (L) found between PdV2 and PdV3.

Overall, three phage peptide variants that bound to rPvAMA1, i.e., PdV1 (DLTFTVNPLSKA), PdV2 (WHWSWWNPNQLT), and PdV3 (TSVSYINNRHNL) were successfully selected from 16 random picked phage isolates in the third round of biopanning with frequency of 8 (50%), 7 (44%), and 1 (6%), respectively. The presence of consensus motifs in the binding peptides with affinity to rPvAMA1 were then investigated using ClustalW (Thompson, Higgins & Gibson, 1994) (Fig. 3).

### Peptides binding assays

**Figure 4:**
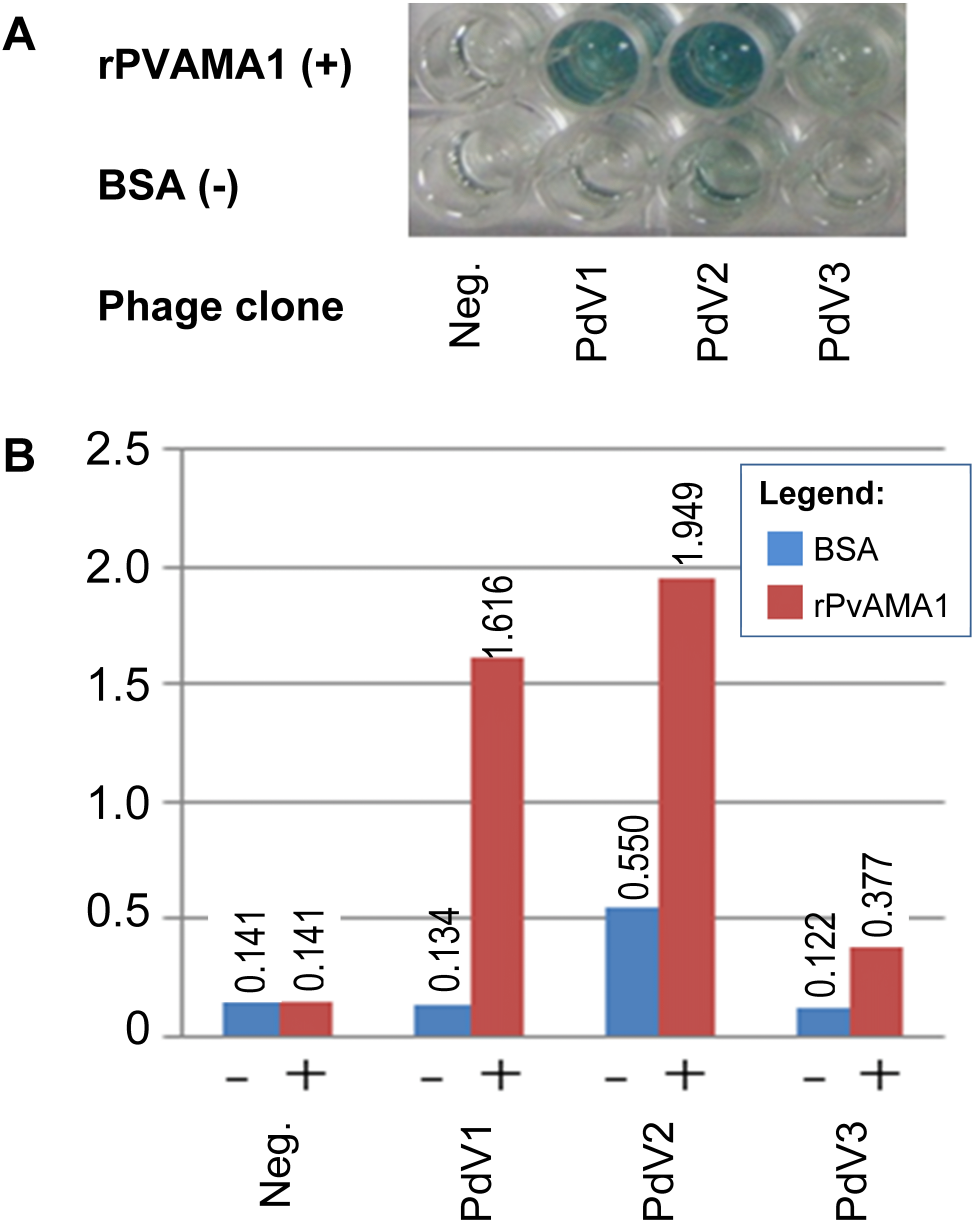
Phage ELISA binding assay for peptides selected from biopanning against rPvAMA1. (A) Upper wells of microplate were coated with 100 µg/ml of rPvAMA1, whereas lower wells were coated with BSA. Negative (Neg.) indicates that none of phage clone was tested. (B) Phage binding signals were determined using HRP-conjugated anti-M13 antibody and ABTS as the HRP-substrate. The binding signals for each peptide were estimated using absorbance value at 405 nm towards BSA (control) and rPvAMA1, respectively.

**Figure 5:**
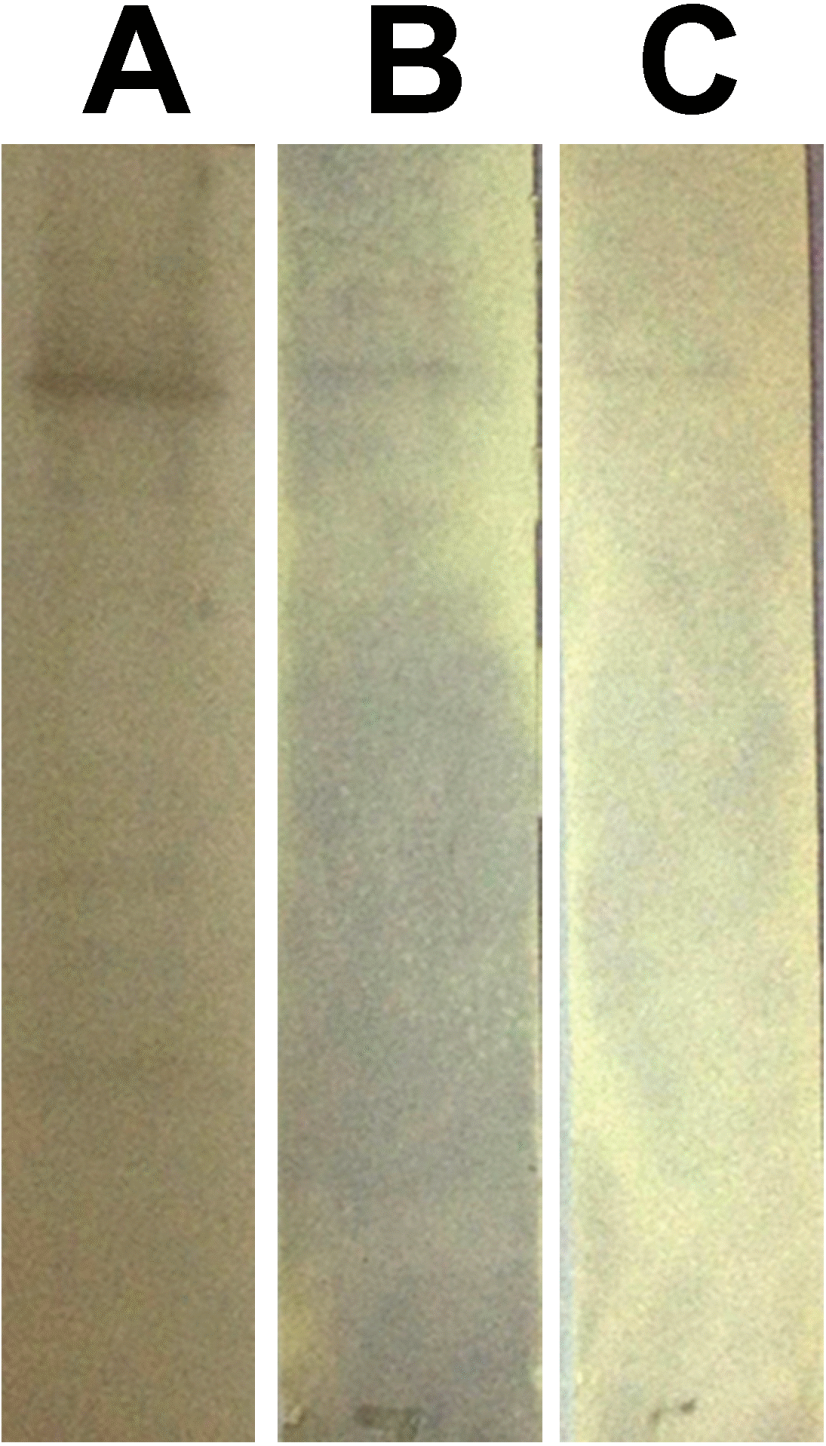
Western blot assay for peptides binding to rPvAMA1. Each individual strips of nitrocellulose membrane (0.45 µm; Millipore, Billerica, MA, USA) blotted with 10 µg rPvAMA1. The binding affinity of the individual phage clone (∼10^9^ phage particles), i.e., PdV1 (A), PdV2 (B), and PdV3 (C) to rPvAMA1 was determined using 1:2,500 HRP-conjugated anti-M13 monoclonal antibody (GE Healthcare, Chicago, IL, USA) and visualized using Opti-4CN (Bio-Rad, Hercules, CA, USA) substrate.

In phage ELISA assay, three phage clones selected from biopanning on rPvAMA1, i.e., PdV1, PdV2, and PdV3 showed positive binding signal with 12.1×, 3.5×, and 3.1×, respectively (Fig. 4). Whilst in Western blot assay, the phage clones PdV1 showed positive signal to rPvAMA1. In contrast, weak binding signals were being observed in strips PdV2 and PdV3 (Fig. 5).

**Figure 6:**
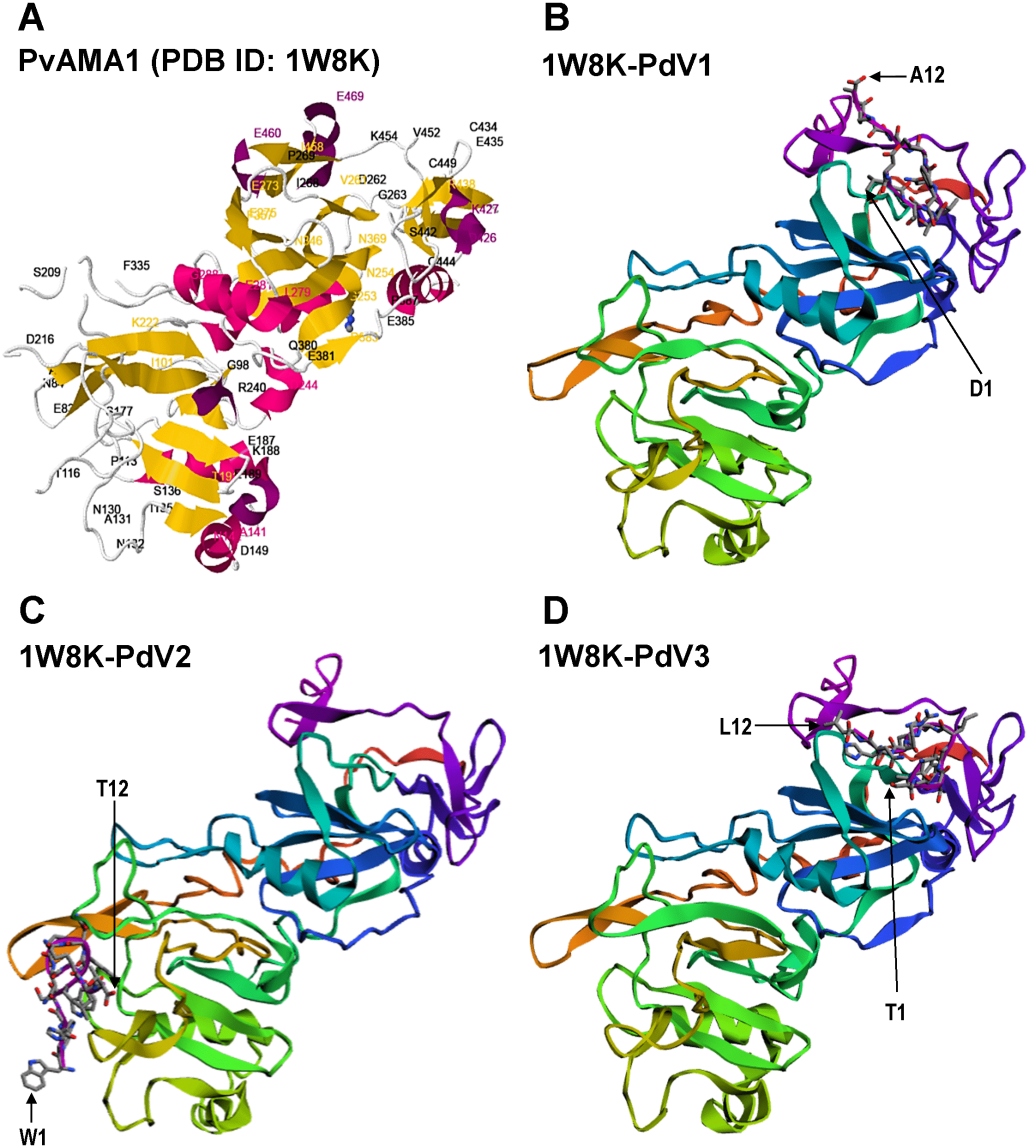
Docking prediction of the binding peptides to the crystal structure of PvAMA1 (PDB ID: 1W8K). The secondary structure PvAMA1 (receptor) are shown in ribbon representation and binding peptides are shown in stick representation. (A) The secondary structure of PvAMA1 (PDB ID: 1W8K) with *β*-strand shown in yellow and *α*-helix in purple. 1W8K-PdV1 complex (B), 1W8K-PdV2 complex (C), and 1W8K-PdV3 complex (D) models are oriented at equivalent angles to 3D structure of 1W8K (A). Arrows point the first and twelfth residues for each binding peptide.

### *In silico* peptide docking

**Figure 7:**
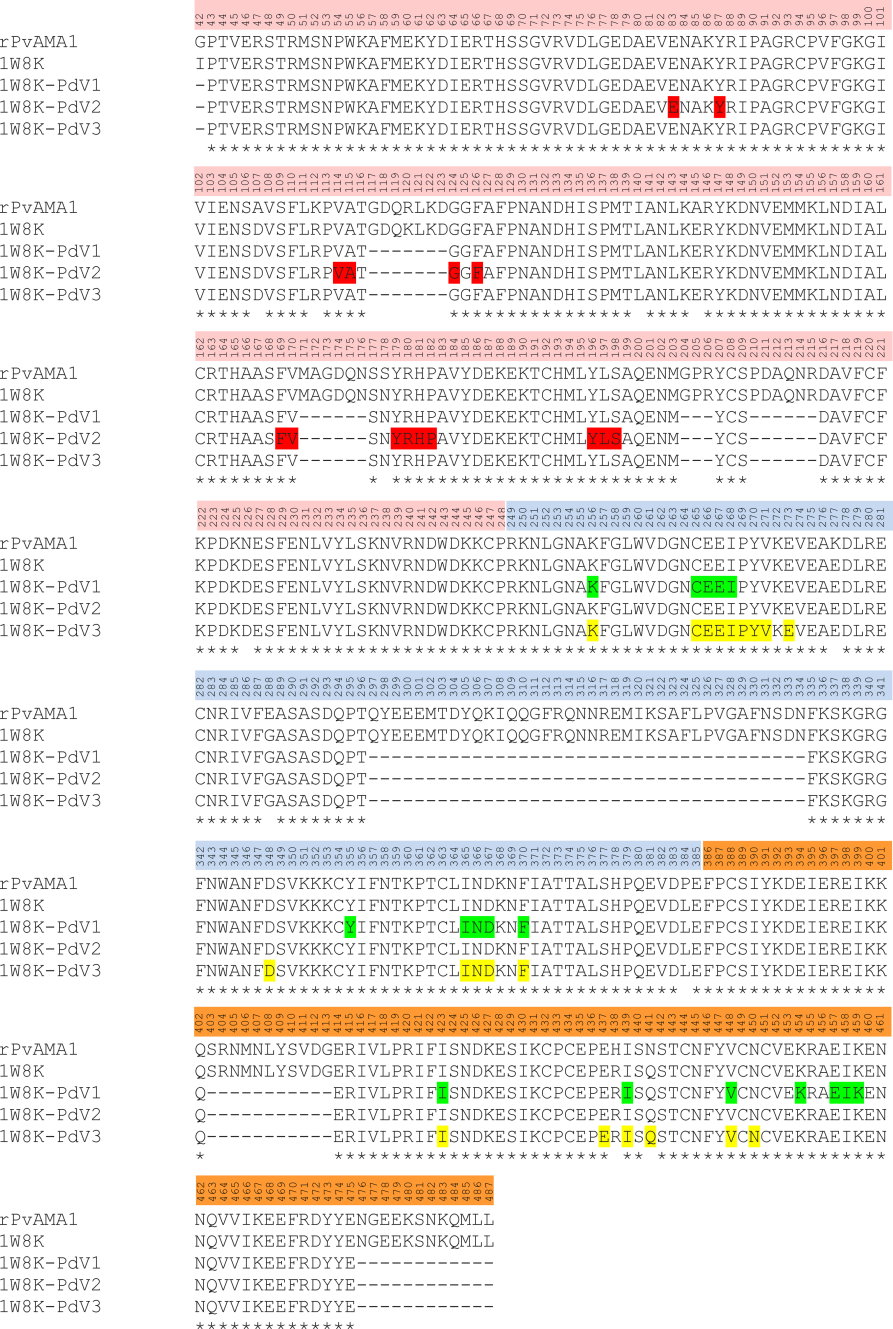
Contact map of the interface between PvAMA1 receptor and peptide closer than 3.5 Å. Amino acid sequences of rPVAMA1 (present study, 100% in concordance with *P. vivax* strain Sal-1, NCBI reference sequence: ACY68841), PvAMA1 (PDB entry 1W8K), and crystal structure sequence of 1W8K encompassing entire ectoplasmic region (residues 43–487) were aligned using ClustalW (Thompson, Higgins & Gibson, 1994), with DI (residues P43-P248) in pink, DII (residues R249-E385) in blue, and DIII (residues F386-L487) in orange. The amino acid residues highlighted with green, red, and yellow represent the predicted binding sites of peptides PdV1, PdV2, and PdV3, respectively closer than 3.5 Å (view detail in Table 1). Main-chain gaps present in 1W8K crystal structure: residues 117–123, 171–176, 204–206, 210–215, 297–334 (DII loop), 403–413, and 476–487. Three amino acid residues of 1W8K were substituted (i.e., S178N, N226D, and N441Q) on putative N-glycosylation sites (NxS/T), adapted to *Pichia pastoris* expression system and amino acid variants present in the residues 107, 112, 140, 145, 277, 288, 384, and 438.

The 3D crystal structure of receptor/peptide binding complex for each peptide docking protocol are oriented at equivalent angles to PvAMA1 (PDB ID: 1W8K) as reference structure (Fig. 6). The predicted pairs of peptide/receptor residues closer than 3.5 Å in the selected complex were listed in Table 1 and the location of each binding site was mapped to a native PvAMA1 (1W8K) amino acid residues (Fig. 7). The simulation models of protein-peptide docking indicated that the PdV1 and PdV3 peptides were mapped to the similar regions, mainly at domains II and III and shared 12 similar binding sites, whereas PdV2 peptide was mapped solely to the DI of PvAMA1 (Fig. 7). Several amino acid variants were detected at residues 107, 112, 140, 145, 277, 288, 384, and 438 among rPvAMA1 (present study) and PvAMA1 (PDB entry 1W8K). In the present study, all peptide binding sites were mapped to the invariant residues of rPvAMA1 and 1W8K sequences, except Q441 for 1W8K-PdV3 complex. Amino acid substitutions on putative N-glycosylation sites (NxS/T) of PvAMA1 (PDB entry 1W8 K), i.e., S178N, N226D, and N441Q are due to the adaptation of the *Pichia pastoris* expression system.

**Table 1:** Pairs of peptide/receptor residues closer than 3.5 Å in the selected complex.

PdV1 (residue)	1W8K (residue)	PdV2 (residue)	1W8K (residue)	PdV3 (residue)	1W8K (residue)
D1	Y355	W1	–	T1	Q441
L2	E266, I268	H2	–	S2	K256, D348, D367, F370
T3	K256, E267, F370	W3	F169, V170, Y179	V3	K256, E267, I365, D367
F4	E267, D367	S4	E83, Y87, Y179	S4	I365, N366, D367, I439
T5	I365, N366, D367, I439	W5	Y196	Y5	C265, I423, V448
V6	C265, I423, V448	W6	Y87, Y179, R180, H181, P182, S198	I6	E437, N450
N7	E266	N7	–	N7	E266
P8	K454	P8	Y87	N8	E266, E267
L9	–	N9	–	R9	E267
S10	I458	Q10	–	H10	E267, I268, P269, Y270, V271
K11	–	L11	V114, F126, L197	N11	–
A12	E457, K459	T12	A115, G124, F126	L12	V271, E273
Total contact pairs	20		20		29

## Discussion and Conclusions

### Expression of recombinant PkAMA1 and PvAMA1 in *E. coli*

A novel Profinity eXact™ fusion-tag prokaryotic expression system (Bio-Rad) was applied in the present study in order to perform heterologous expression of recombinant PvAMA1 ectodomainin in BL21-CodonPlus (DE3)-RIL *E. coli* cells (Strategene, La Jolla, CA, USA), suited for heterologous expression of recombinant protein consisted of AT-rich genome such as *Plasmodium* species. Profinity eXact fusion-tag system is an affinity tag-based protein purification system that utilizes a modified form of the subtilisin protease, which is immobilized onto a chromatographic support. The unique feature of this system is that it allows a single step affinity purification and on-column fusion-tag cleavage of recombinant protein to produce pure, native protein (tag-free target protein). We postulated that the expression of tag-free native form of rPvAMA1 may aid in the more precise biopanning activity.

Generally, proteins expressed in recombinant manner may be soluble in the cytoplasm or insoluble as IBs, which may affect the subsequence protein extraction method to obtain the starting material for purification. Soluble protein is thought to be native in structure and function, while insoluble aggregated protein reflects improper fold and lack of functionality. The eukaryotic proteins intracellularly expressed in *E. coli* system are frequently sequestered into insoluble IBs. The intramolecular associated to the hydrophobic domains during folding is believed to play a role in the formation of IBs. Furthermore, for proteins with high cysteine residues like AMA1, improper formation of disulfide bonds in the reducing environment of *E. coli* cytoplasm may also contribute to incorrect folding and formation of IBs. Reducing SDS-PAGE and Western blot assays showed that pPAL7-PvAMA1 was deposited in insoluble fraction with molecular weight appearance of ∼60 kDa. This made it necessary to add an additional IB purification step after cell disruption prior to affinity purification. Generally, IBs often contain exclusively (40%–90%) the overexpressed protein and successful in renaturation depends much on the purity of IBs (Cabrita & Bottomley, 2004). To date, many protocols have been established to overcome the problems due to IB formation. Generally, three main steps are required to recover the functional active protein from IB: first step is the isolation and purification of IB from *E. coli* cells; second step is the solubilization of aggregated protein or purified IB, which causes denaturation; and finally refolding to renature the denatured protein.

The Profinity eXact system manufacturer’s protocol is only available for purification of protein under native and soluble conditions and therefore in our present study, we optimized the purification protocol under denatured and reduced conditions, which means all buffers used must keep the protein in the denatured reduced stage during the procedure. The concentrations of urea ranged from 2 M to 8 M (not reported here) were optimized and 4 M urea was sufficient to solubilize the IB. In the present study, tag-free rPvAMA1 was successfully isolated from purified and solubilized IB under denatured conditions. The removal of Profinity eXact tag with molecular mass of ∼8.2 kDa generated rPvAMA1 of high purity and integrity with a molecular weight of ∼51 kDa as concordance to the estimated size using ExPASy tool (Fig. 1). LC-MS/MS data of rPvAMA1 hit two crystal structures of AMA1 from *P. vivax* with NCBI accession number 62738184 (PBD ID: 1W81_A) and 62738193 (PBD ID: 1W8K_A). Establishment of purification protocol under denatured conditions may enhance the utilities of this protein expression system that allows rapid single affinity purification and tag-removal.

#### Renaturation of the rPvAMA1

The correct folding of eight disulfide bonds within AMA1 ectodmain has been shown to be critical for its immunological activity. Only the antibodies raise against refolded AMA1 inhibited parasite growth *in vitro*. In contrast, the reduced and alkylated AMA1 failed to induce protective immunity. Furthermore, this irreversible reduced AMA1 was not recognized by antibodies raised against the native antigen, and thus suggest that an effective immune response is much dependent on conformational epitopes maintained by a set of eight disulfide bonds (Lalitha et al., 2004; Pizarro et al., 2005). For the above mentioned reasons, the present study therefore, included the protein renaturation step that was aimed to restore the function of rPvAMA1, which was expressed in the form of insoluble IB and purified under denatured conditions.

Generally, extracellular domains of malaria antigens, such as AMA1 almost invariably contain disulfide linkages, thus production of this kind of proteins in the native conformation remains particularly difficult (Flick et al., 2004). Disulfide bonds are derived by the coupling of two thiol groups (two cysteine residues) to form a covalent disulfide (S-S) bond, which can be intra- or inter-molecular bridges. Native AMA1 ectodomain is high-cysteine protein, consists of 16 cysteine residues that are responsible for eight pairs of invariant intramolecular disulfide bonds, and this undoubtedly increase the difficulty in protein renaturation process. The renaturation process aims to effectively remove the denaturant and allow the protein to fold (Cabrita & Bottomley, 2004).

To date, there are many successful expression and refolding of *Plasmodium* AMA1 ectodomain which have been published elsewhere, of which most were of PfAMA1 (Hodder, Crewther & Anders, 2001; Dutta et al., 2002; Kocken et al., 2002; Lalitha et al., 2004; Gupta et al., 2005) and a few in PvAMA1 (Pizarro et al., 2005; Mufalo et al., 2008). The REFOLD database (http://refold.med.monash.edu.au/) is a good platform that summarizes all established refolding protocols, including AMA1 protein (Chow et al., 2006). Present refolding protocol was performed by dialysis method, whereby the concentrated denatured rPvAMA1 (1 mg/ml) was dialyzed against two changes of refolding buffer (at two progressive lower denaturant concentrations, i.e., 2 M and 1 M urea). Such that, the concentration of denaturant decreases with buffer exchange under step-wise manner, followed by last changing to sample buffer with the absence of urea which is compatible for biopanning.

The simple way to monitor the refolding condition is by direct visual inspection of the turbidity of solutions, where protein aggregation has occurred. The formation of insoluble aggregates is an indication of non-optimize in refolding protocol. The turbidity of the sample solution also can be assessed more accurately by measuring the optical density (OD) at 390 nm of the refolded protein solution (Vincentelli et al., 2004). Generally, the solution is not absorbing the light, but rather the protein aggregates scatter light and thus decrease the amount of transmitted light measured. In other words, OD remains unchanged if the protein remains soluble, in contrast, the OD increases proportionally to the amount of precipitated produced. There were no insoluble aggregates being observed in rPvAMA1 protein solutions after refolding. Furthermore, the protein OD_390_ in the turbidity test was less than 0.05 in rPvAMA1 protein solutions, suggesting that refolded AMA1 protein was soluble. The reduced and non-reduced SDS-PAGE results also supported that rPvAMA1 was refolded.

### Phage display technology and applications in Plasmodium AMA1

Phage display is a powerful and cost effective tool, which is widely used for the investigation of protein-protein interactions, immunoassay development, identification of peptide agonists and antagonists for receptors, identification of targets for the inhibition, elucidation of neutralizing sites, investigation into pathogenesis of disease, identification of peptide drug and vaccine candidates, and the isolation and engineering of recombinant antibodies (Wang & Yu, 2004; Mullen et al., 2006; Lanzillotti & Coetzer, 2008). Studies in protein-protein interactions might contribute to some exploration of molecular function of plasmodia proteins thus enhance the development of novel therapeutics. The phage display library is an assembly of peptides library which consists of billions of short and variable amino acid sequences displayed on the surfaces of bacteriophage M13. The peptide library allows the selection of peptides, which have high binding affinity to the immobilized target protein. Previous phage display studies on AMA1 protein are mainly focused on *P. falciparum* and there is a vital need to include studies based on other significant species, such as *P. vivax*.

To date, protective responses against the *Plasmodium* AMA1 have been investigated using various phage display libraries. For instance, four antibodies specific for *Plasmodium* AMA1 have been identified by mouse single chain variable fragment (scFv) antibodies library (derived by *P. chabaudi* immunized mouse) and were used for passive immunization against *P. falciparum* AMA1 (Fu et al., 1997). Most likely antigenic peptides were obtained from epitope mapping, in which direct antibody ELISA is used to affinity select antigenic peptides from very large libraries of peptides displayed on filamentous phage carries. If natural peptide library is used in selection, antigenic peptide is called epitope, while mimotope is selected from the random peptide library. Epitope mapping using PfAMA1 peptide library (Coley et al., 2001; Coley et al., 2006) as well as mimotopes mapping using random peptide library (Casey et al., 2004; Sabo et al., 2007) against monoclonal anti-AMA1 antibodies (e.g., 5G8, 4G2dc1, and 1F9) that blocks merozoite invasion by *P. falciparum* have facilitated greater understanding of immune responses against AMA1. Several peptides that specifically bind to PfAMA1 and showed similar functionality to native anti-AMA1 have been isolated from random peptides libraries (Li et al., 2002; Harris et al., 2005). All of these binding peptides and their analogues were synthesized and further validated with antibody competition ELISA assay as well as growth inhibition assay and immunofluorescence assay based on parasite cell culture (Keizer et al., 2003; Harris et al., 2009).

In some circumstances, peptides with high affinity to the target antigen can also be used in drug-discovery process, although these peptides themselves do not generally make good drugs, they can provide a backbone for the peptidomimetic design of efficient drugs (Barbas et al., 2001). As example, the structure of a phage displayed 15-residue peptide, F1 against PfAMA1 (Li et al., 2002) provided a valuable starting point for the development of peptidomimetic as antimalarial antagonists directed at AMA1 (Keizer et al., 2003). Most recently, one of the most prominent peptide inhibitor, a 20-residue peptide designated as R1 appears to target a site critical for PfAMA1-RON2 function and subsequently blocks the parasite invasion has been modified to be a potent leading compound for antimalarial drug development (Harris et al., 2005; Harris et al., 2009; Wang et al., 2014).

Nowadays, applications of both phage display technique together with the 3D structuring and/or protein docking techniques have sped up the studies on molecular binding interactions. For instance, the AMA1-binding molecules, i.e., monoclonal antibodies (both the epitopes and mimotopes) and peptides selected from phage display libraries, all of which block merozoite invasion of erythrocytes, were successfully characterized and have been mapped to the location on AMA1 ectoplasmic region by nuclear magnetic resonance spectroscopy (Keizer et al., 2003; Sabo et al., 2007; Harris et al., 2009; Wang et al., 2014) and X-ray crystallographic analysis (Bai et al., 2005; Pizarro et al., 2005). Overall, all the studies described above focused on PfAMA1 and this is the first study carried out on PvAMA1.

### Identification of peptides affinity for PvAMA1

From the successful and promising results obtained from the previous studies on the identification of binding peptides for PfAMA1 as mention above, the objective of the current study was to identify novel and potential binding peptides that have high binding affinity to PvAMA1. Differing from the previous studies, a ready-made library of random 12-residue peptides expressed as N-terminal fusion to protein III of filamentous phages M13 was used for identification of binding peptides for rPvAMA1. In the present study, three dodecapeptide variants that have binding affinity to refolded rPvAMA1 were identified through the Ph.D.-12 random peptide library (NEB). The biopanning process often generates peptides with conserved consensus sequences, which can then be chemically synthesized on the basis of consensus sequences and are evaluated by bioassay and structural analysis (Uchiyama et al., 2005). The selected peptides (synthetic forms) can be used to antagonize the interactions between two particular proteins, which may have an effect on the biological activities of the target protein. Alternatively, these peptides may also have an effect on of the activity of enzyme, either through active site inhibition or long-range interactions (Kay, Kasanov & Yamabhai, 2001). There were a lack of consensus of sequence motif found amongst binding peptides selected from biopanning against rPvAMA1 (PdV1, PdV2, and PdV3). Our present observations were similar to two previously done biopanning against PfAMA1 that also aimed to identify the binding peptides (Li et al., 2002; Harris et al., 2005). A follow up of these studies indicated that these binding peptides were mapped to different domains of PfAMA1 with different binding activities using NMR spectroscopy (Keizer et al., 2003; Harris et al., 2009) and it was reasonable to explain the lack of consensus sequence motif in such kind of study.

The binding affinity of each phage peptide variants to rPvAMA1 was further determined using phage ELISA as well as Western blot binding assays. Three peptides, i.e., PdV1 (DLTFTVNPLSLA), PdV2 (WHWSWWNPNQLT), and PdV3 (TSVSYINNRHNL), identified from biopanning against rPvAMA1 showed 12.1-fold, 3.5-fold, and 3.1-fold binding signal in phage ELISA binding assay, respectively. Even though, the peptide PdV2 showed the highest binding signal among three peptides, however the background binding was also quite high and thus lowered the fold of binding signal. In Western blot assay, sharp and clear binding signal was observed in PdV1, while weak binding signal was observed in peptides PdV2 and PdV3. Based on the preliminary data obtained from the present study, it was hard to make a complete conclusion on these individual peptides with affinity to rPvAMA1.

### *In silico* peptide docking

Identifying functionally critical regions such as peptide ligands as well as ligand- or receptor-binding sites that specifically interact with and block the function of AMA1 is essentially important to facilitate the design of an effective vaccine. The X-ray crystallography is a gold standard method that allows direct 3D view of the interaction between the two compounds such as antigen and antibody. However, this method is time-consuming, expensive, and technically challenging, requiring a large amount of homologous and highly purified native protein. *In silico* protein docking undoubtedly overwhelming these limitations by allowing simulation study of the target protein-peptide interactions utilizing currently available protein structure databases (PDB). For instance, CABS-dock is a free web server for the flexible docking of peptides to target protein without prior knowledge of the binding site and also allows prediction of complex arrangements close to the native structure (Kurcinski et al., 2015; Blaszczyk et al., 2016). Furthermore, the major obstacle to the development and use of functional assays for *P. vivax* is the difficulty of cell culturing and long term maintenance of the parasite *in vitro*. Generally, *P. vivax* shows preference to young blood type, i.e., reticulocytes. This characteristic makes it very difficult to manipulate in the laboratory, which has held back progress on vaccine and therapeutic developments targeting *P. vivax*. In the present study, the binding sites of the phage display selected peptides toward PvAMA1 were therefore predicted using *in silico* protein-peptide docking freeware.

A greater understanding of the molecular interaction between the parasite and its host would assist in the development of new therapeutics and most importantly, a vaccine for long term sustainable reduction in the global burden of malaria. It has become clearer that immunodeterminant regions are dispersed entirely on the ectodomian of AMA1, i.e., DI, DII, and DIII. For instance, the phage display derived R1 peptide binds AMA1 competitively with mAb 1F9 and the IgNAR, as well as mAb 4G2, suggesting that R1 interacts or share similar epitope near to hydrophobic groove of DI and flexible region of DII loop, respectively (MacRaild et al., 2011). Crystal structure studies demonstrated that the mAb R31C2 binds to both hydrophobic groove of DI and DII loop (Vulliez-Le Normand et al., 2015), whereas mAb F8.12.19 recognizes discontinuous epitope located on DIII (Igonet et al., 2007) and all of the mentioned monoclonal antibodies and R1 peptide block merozoite invasion of erythrocytes *in vitro*.

In the present study, the pairs of peptide/receptor residues closer than 3.5 Å for 1W8K-PdV1 and 1W8K-PdV3 complexes were mapped within domains II and III with sharing 12 similar binding residues even though these two peptides did not show any consensus sequence (Fig. 3). Majority of the PdV1 and PdV3 binding sites were mapped at or closer to cysteine residues of domains II and III, whereby disulfide bonds cross linked, i.e., C265-C363 and C282-C354 at DII and C388-C444, C432-C449, and C434-C451 at DIII, responsible for the structural and functional stability (Pizarro et al., 2005). The present binding peptides were selected from the refolded rPvAMA1, we therefore postulated that PdV1 and PdV3 are able to bind to native PvAMA1 in nature and thus offer a good alternative as antimalarial drug delivery compound. Furthermore, some of binding sites were dispersed closed to epitope recognized by mAb F8.12.19 (34-residue segment of DIII from I421-K454) (Igonet et al., 2007). In contrast to peptides PdV1 and PdV3, the PdV2 peptide binding sites were mapped solely to DI. Domains I and II belong to the plasminogen-apple-nematode (PAN) module superfamily, which are found in proteins with diverse adhesion functions by mediating protein-protein and protein-carbohydrate interactions (Pizarro et al., 2005). In general, none of the peptide residues were *in silico* docking to the seven main-chain gaps regions or disorder regions that could not be built to the electron density. Surface-protruding loops that lack of regular secondary structure are most pronounced in DI (several in DI, one in DII and one in DIII) (MacRaild et al., 2011). Interestingly, we notice that there were two continuous receptor/peptide binding hot spots of PdV2 flanking across two terminal regions of main-chain gaps at residues 117–123 and 171–176. Even though very hard to come out with any conclusion regarding this binding motif, from the previous study, the loop regions of AMA1 are likely to be functionally significant. For instance, linear B cell epitope are located at S290-K307 of PvAMA1, in line to the DII loop (residues Q297-N334), a binding site for RON2 during parasite host cell invasion. In conclusion, in depth understanding of the binding peptides provide a valuable starting point for the development of peptidomimetic as antimalarial antagonists directed at PvAMA1.

## Additional Information and Declarations

### Data Availability

The following information was supplied regarding data availability: The raw data is included in the manuscript.
